# Effect of two weeks of training cessation on concentric and eccentric knee muscle strength in highly trained sprinters

**DOI:** 10.1371/journal.pone.0288344

**Published:** 2023-07-07

**Authors:** Daichi Yamashita, Kosuke Hirata, Kazuhiko Yamazaki, Iñigo Mujika, Naokazu Miyamoto

**Affiliations:** 1 Department of Sport Science and Research, Japan Institute of Sports Sciences, Tokyo, Japan; 2 Faculty of Sport Sciences, Waseda University, Saitama, Japan; 3 Faculty of Health and Sports Science, Juntendo University, Chiba, Japan; 4 Department of Physiology, Faculty of Medicine and Nursing, University of the Basque Country, Leioa, Basque Country; 5 School of Kinesiology, Faculty of Medicine, Universidad Finis Terrae, Santiago, Chile; Universidad de León Facultad de la Ciencias de la Actividad Física y el Deporte: Universidad de Leon Facultad de la Ciencias de la Actividad Fisica y el Deporte, SPAIN

## Abstract

Athletes often experience short-term training cessation because of injury, illness, post-season vacation, or other reasons. Limited information is available about the effect of short-term (less than four weeks) training cessation on muscle strength in athletes. Sprinting athletes must maintain knee extension and flexion strength to reduce the risk of sprint-type hamstring strain injury. This study aimed to identify whether and to what extent knee extension and flexion torque in concentric and eccentric contractions is reduced by two weeks of training cessation in sprinters. Before and after the training cessation, maximal voluntary isokinetic knee extension and flexion torque in slow and fast concentric (60 and 300°/s) and slow eccentric (60°/s) contractions were assessed in 13 young male highly trained sprinters (average World Athletics points = 978). Knee flexion torque during the bilateral Nordic hamstring exercise (NHE) was also measured. After the training cessation, isokinetic concentric at 300°/s and eccentric torque were significantly reduced in both knee extension and flexion. There was no difference in the magnitude of reduction between isokinetic knee extension and flexion torques in all conditions. The relative changes were more notable in eccentric (-15.0%) than in concentric contraction at 60°/s (-0.7%) and 300°/s (-5.9%). Knee flexion torque during the NHE also declined (-7.9% and -9.9% in the dominant and non-dominant legs, respectively). There was no significant correlation between the relative reductions in isokinetic knee flexion torque and knee flexion torque during the NHE. The findings suggest that sprinters and their coaches should focus on recovering fast concentric and slow eccentric knee extension and flexion strength after two weeks of training cessation.

## Introduction

Athletes often experience training cessation because of injury, illness, post-season vacation, or other reasons. The magnitude of a training cessation-induced decline in their physical functions strongly depends on the duration of training cessation and/or the training level of subjects [1–3]. Regarding muscle performance, to date, much attention has been paid to notable declines in muscle strength after long-term training cessation (i.e., longer than four weeks) [2]. In contrast, limited information is available, and a scientific consensus has not yet been reached on the effects of short-term (i.e., less than four weeks) training cessation on muscle performance in power-trained athletes [1]. In particular, there is little evidence for the effects of approximately two weeks which often correspond to the time of return to training or play after mild-to-moderate injuries, seasonal break in a league [4], or the period of home confinement imposed by the COVID-19 pandemic as observed around the world [5]. An advanced understanding of these unknown consequences can provide practitioners with a basis for developing more effective training programs in return to training or playing after such periods of training stoppage.

A few studies have examined the effect of approximately two weeks of training cessation on concentric or eccentric muscle strength in athletes [6–8]. However, the findings are inconsistent. In power-trained athletes (powerlifters and American footballers), a significant decrease was reported in isokinetic eccentric knee extension strength after 14 days of training cessation, but not in isometric and isokinetic concentric knee extension strength [7]. Surprisingly, no significant changes in knee flexion strength were found regardless of contraction types (isometric, concentric, or eccentric). In endurance athletes (long-distance runners, triathlon, and marathon athletes), a significant decrease was observed in concentric knee extension strength but not in concentric knee flexion strength [6]. In soccer players, isokinetic concentric strength remained unchanged for both knee extension and flexion after two weeks of training cessation [8]. Several studies have reported lower neuromuscular activation during eccentric contractions of knee extension than during isometric contractions [9–11]. Additionally, neural adaptations induced by resistance training are greater in eccentric than concentric contractions [12]. Based on these findings, it is reasonable to consider that eccentric strength is more susceptible to detrimental effects of neuromuscular activation after short-term training cessation than concentric strength. However, the effect of short-term training cessation on concentric and eccentric strength of knee extension and flexion may depend, at least in part, on the training background and specialization of the subjects. For example, knee flexion strength, while being important, might not be as crucial for powerlifters, long-distance runners, and soccer players as it is for sprinters. In contrast, knee flexion strength is crucial to superior sprint performance [13–15], and eccentric knee flexion strength is considerably higher in sprinters than in athletes of other field sports [16]. Thus, whether knee flexion strength is unaffected by short-term training cessation should be ascertained in highly trained sprinters for whom knee flexion strength plays a crucial role.

For the prevention of hamstring strain injury, declined knee flexion strength must be precluded in sprinting athletes because weak isokinetic eccentric knee flexion strength is a risk factor for a sprint-type hamstring strain injury [17, 18]. Recently, the Nordic hamstring exercise (NHE) has also been performed to prevent hamstring strain injuries and used to assess their risks [19–22]. Both the isokinetic knee flexion and Nordic hamstring exercise tests are commonly employed to evaluate eccentric knee flexion strength. However, there is only a weak correlation between the values obtained from each test [22], indicating that they may assess different characteristics of the knee flexors. Thus, from the viewpoint of injury prevention, the effect of short-term training cessation on eccentric knee flexion strength should be examined not only using an isokinetic dynamometer but also during the NHE. Therefore, the present study aimed to examine the effects of a two-week training cessation on changes in knee extension and flexion strength, measured in an isokinetic dynamometer test of concentric and eccentric contractions, and in the NHE test, in sprinters. We hypothesized that the magnitude of decline in muscle strength would differ between contraction types and that eccentric strength is more susceptible to two weeks of training cessation than concentric strength in both knee extension and flexion in sprinters.

## Materials and methods

### Subjects

Thirteen highly-trained male sprinters (age = 20.4 ± 1.4 years, height = 174.1 ± 4.5 cm, body mass = 65.6 ± 4.4 kg, experience of competitions: 6.8 ± 2.0 years) participated in this study. The inclusion criteria were (i) current participation in competitive races and (ii) at least three years of regular training in track sprinting. The exclusion criterion included any individuals with lower extremity injuries or illness that would prevent them from regular training at the time of the pre-test, or those who intended to engage in training, even for a single day, during before the two-week training cessation period. The G*Power version 3.1.9.2 (Heinrich-Heine-Universität Düsseldorf, Düsseldorf, Germany) was used to determine the appropriate sample size before this study. For a two-way repeated-measures analysis of variance (ANOVA), we set power at 80% with 5% error level and the effect size at 0.25 (medium) and found that at least 12 participants were required to meet these criteria. The World Athletics points for the season-best time of the specialized event of each subject ranged from 790 to 1200 (978 ± 111). Based on the classification framework proposed by McKay et al. [23], the subjects were categorized as three Tier-2 (trained/developmental), nine Tier-3 (highly trained/national level), and one Tier-4 (elite/international level). The dominant leg in the present study was defined as the preferred jumping leg [24]. Six participants were left-dominant, and seven participants were right-dominant. Prior to participation, all subjects were fully informed of the purpose, experimental procedures, and their right to withdraw from the study at any time without penalty, and gave their written consent to participate in the experiment. Our hypotheses were not explained to the subjects to exclude any potential bias that might affect the results. This study was approved by the institutional ethics committee (No. 2021–055) and performed in accordance with the Declaration of Helsinki.

### Experimental procedure

The two weeks of training cessation started immediately after a competitive season. Isokinetic knee extension and flexion torque, knee flexion torque during the NHE, and total and segmental body composition were assessed before and after two weeks of training cessation. During the two weeks, the subjects were instructed to maintain their normal daily activities but not to engage in any physical training, including resistance exercises and stretching exercises (although this was not monitored). All tests were performed at the same time of day for each subject. The subjects were instructed to avoid drinking and exercising for two hours prior to testing (although this was not monitored). During test familiarization before each test, subjects were provided with the relevant test instructions and time to practice each test to ensure they were familiar with all procedures.

### Body composition

Body composition measurement was performed prior to all muscle strength testing to avoid any influence of fluid shift, such as exercise-induced muscle swelling. Before the measurement, the subjects lay supine for 10 min to avoid the influence of whole-body fluid shift due to standing [25]. Total body mass, percent body fat, and muscle mass of total body and each leg were measured by a bioelectrical impedance device (Inbody 730, Biospace, Japan). All subjects wore minimal, light clothing free of metallic material.

### Isokinetic knee extension/flexion

Subjects were seated on an isokinetic dynamometer (CON-TREX MJ, CMV AG, Switzerland) with the hip flexed at 80° (supine lying position = 0°). The thorax, pelvis, and thigh were tightly secured to the dynamometer seat using non-elastic straps. The lever arm of the dynamometer was attached 3–4 cm above the lateral malleolus. The rotation axis of the dynamometer was aligned with that of the knee. Due to the time constraint of the present experiment, isokinetic knee extension/flexion torque was measured only on the right leg for all subjects. However, the limitation should not affect the results of this study because a previous study reported no significant differences in peak isometric torque (60°/s) of the hamstrings and quadriceps between the dominant and non-dominant leg for elite sprinters [26]. An adequate familiarization with the dynamometer was provided as a form of warm-up isokinetic exercises (at least one repetition with each of 20%, 50%, and 80% efforts). The testing protocol consisted of 3 sets of consecutive repetitions of knee extension and flexion with maximal efforts. The first and second sets were slow and fast isokinetic concentric contractions at angular velocities of 60°/s (CON60; 3 repetitions each for knee extension and flexion) and 300°/s (CON300; 3 repetitions each), respectively. Afterward, subjects performed the last set of slow isokinetic eccentric contractions at an angular velocity of 60°/s (ECC60; 3 repetitions each). Each set of testing was performed with at least 1-min rest provided between the sets [27]. The knee range of motion for each repetition was 95° [110° to 15° (full knee extension = 0°)]. No pre-activation was allowed before contractions. Verbal encouragement was provided, but any visual feedback was not given to the subjects. The torque was sampled at 1 kHz (PowerLab16/35, ADInstrument, Australia). For each knee extension and flexion in any contraction or angular velocity mode, the highest peak torque of the three repetitions was used for further analyses. The within-session (i.e., three repetitions) reliability for each peak torque was considered excellent [28], with intraclass correlation coefficients (ICC_2,1_) = 0.910–0.942.

### Nordic hamstring exercise (NHE)

**Fig 1:**
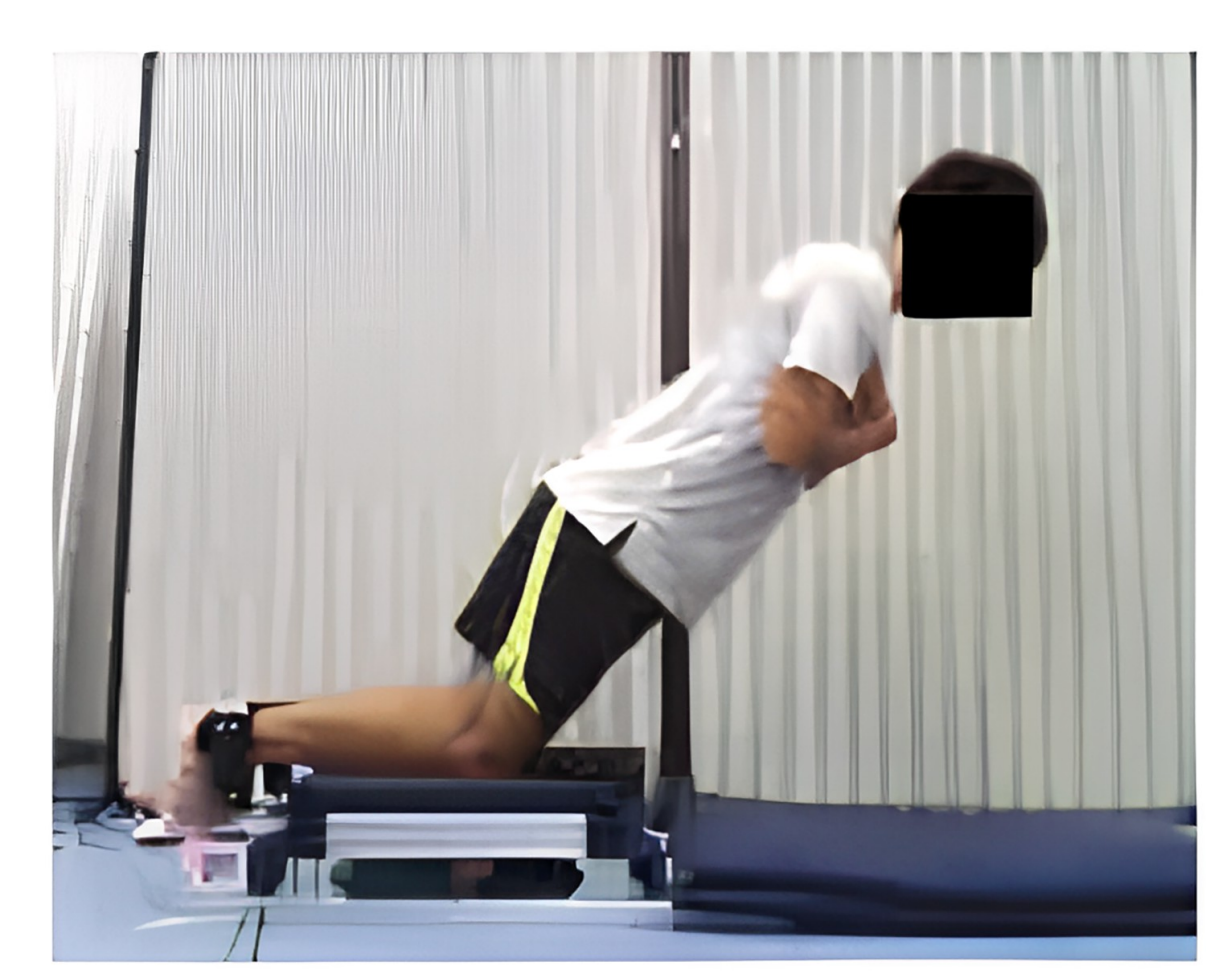
Experimental set-up for assessing Nordic hamstring exercise.

The bilateral NHE was performed with a custom-made device (Fig 1). The subjects kneeled on the padded board of the device. For each leg, a non-elastic ankle brace fixed to a uniaxial load cell (LUX-B-1KN-ID, Kyowa, Japan) was secured immediately above the lateral malleolus. The load cells were positioned perpendicular to the shanks. After warm-up exercises of submaximal repetitions, the subjects performed one set of three maximal repetitions of the bilateral NHE. The subjects were instructed to progressively lower the torso as far as possible at a constant knee extension angular velocity of 18°/s (5 s per 90°) [29], while keeping the trunk and the hips in a neutral position with the hands held across the chest. None of the subjects were able to accomplish near-full knee extension during the NHE. The use of the hands and arms was only allowed to stop the fall and return to the kneeling position. Force data from each leg were sampled at 1 kHz (PowerLab16/35, ADInstrument, Australia). The distance between the lateral epicondyle of the femur and the middle of the strap at the ankle was measured to convert force (N) to torque (N·m) during knee flexion. The peak knee flexion torque for each leg during the NHE was determined in the trial of the highest sum of knee flexion torque for both legs and used for further analyses. The within-session reliabilities for left and right leg were considered excellent, with ICC_2,1_ of 0.902 and 0.938, respectively.

### Statistical analysis

For all parameters, the normal distribution of the data was confirmed using the Shapiro–Wilk test. Paired t-tests were performed to compare before and after the two-week training cessation. Paired t-tests were also used to compare the changes in muscle mass and knee flexion torque during the NHE between the right and left legs and between the dominant and non-dominant legs. To determine whether significant differences in the magnitude of changes in isokinetic knee extension and flexion torque exist between CON60, CON300, and ECC60, a two-way analysis of variance (ANOVA) [direction (extension, flexion) × condition (CON60, CON300, ECC60)] with repeated measures was performed. When appropriate, one-way ANOVA with a Bonferroni post-hoc test was used to determine whether significant differences exist between CON60, CON300, and ECC300. To describe the correlation between eccentric knee flexion strength measured during the isokinetic and NHE, the Pearson correlation coefficient was calculated between peak torque for ECC60 and NHE in the pre-test, post-test, and between the relative changes in peak torque for ECC60 and NHE. The statistical analyses were performed using statistical software (SPSS Statistics Ver. 26, IBM Japan, Japan). The significance level was set at P = 0.05 with Bonferroni correction for multiple comparisons. Descriptive data are expressed as means and standard deviations. Partial eta squared (η^2^) effect sizes were calculated for all ANOVAs and partial η^2^ < 0.06 was considered small, 0.06 to 0.15 medium, and 0.15 < η^2^ large [21]. The Cohen’s d for repeated measures was calculated as an effect size index for the mean comparisons [30]. A |d| < 0.2 was considered trivial, 0.2 to 0.49 small, 0.5 to 0.79 medium, and 0.8 < |d| large [31].

## Results

Total body mass, percent body fat, and muscle mass of total body and each leg before and after the two weeks of training cessation are shown in Table 1. There were no significant changes for any of the variables, except for the segmental muscle mass of the left and non-dominant leg, which was significantly increased with trivial effect sizes after the training cessation (P = 0.020, d = 0.055 and P = 0.026, d = 0.053, respectively).

**Table 1:** Body composition before and after two weeks of training cessation.

	Before	After	95% CI of difference		P value	Cohen’s d
			Lower	Upper		
Total body mass (kg)	65.6 ± 4.4	65.4 ± 4.1	-0.60	0.11	0.154	0.048
Percent body fat (%)	11.7 ± 3.1	11.4 ± 2.9	-1.06	0.37	0.315	0.100
Total muscle mass (kg)	33.3 ± 2.8	33.2 ± 2.7	-0.35	0.32	0.923	0.037
Leg muscle mass (kg)						
Left	9.06 ± 0.75	9.10 ± 0.75	0.01	0.12	**0.020**	0.054
Right	9.07 ± 0.76	9.09 ± 0.76	-0.01	0.10	0.110	0.027
Dominant (Left/Right = 6/7)	9.06 ± 0.75	9.09 ± 0.74	-0.01	0.10	0.096	0.041
Non-dominant	9.07 ± 0.76	9.11 ± 0.77	0.01	0.12	**0.026**	0.053

CI: confidence interval

**Fig 2:**
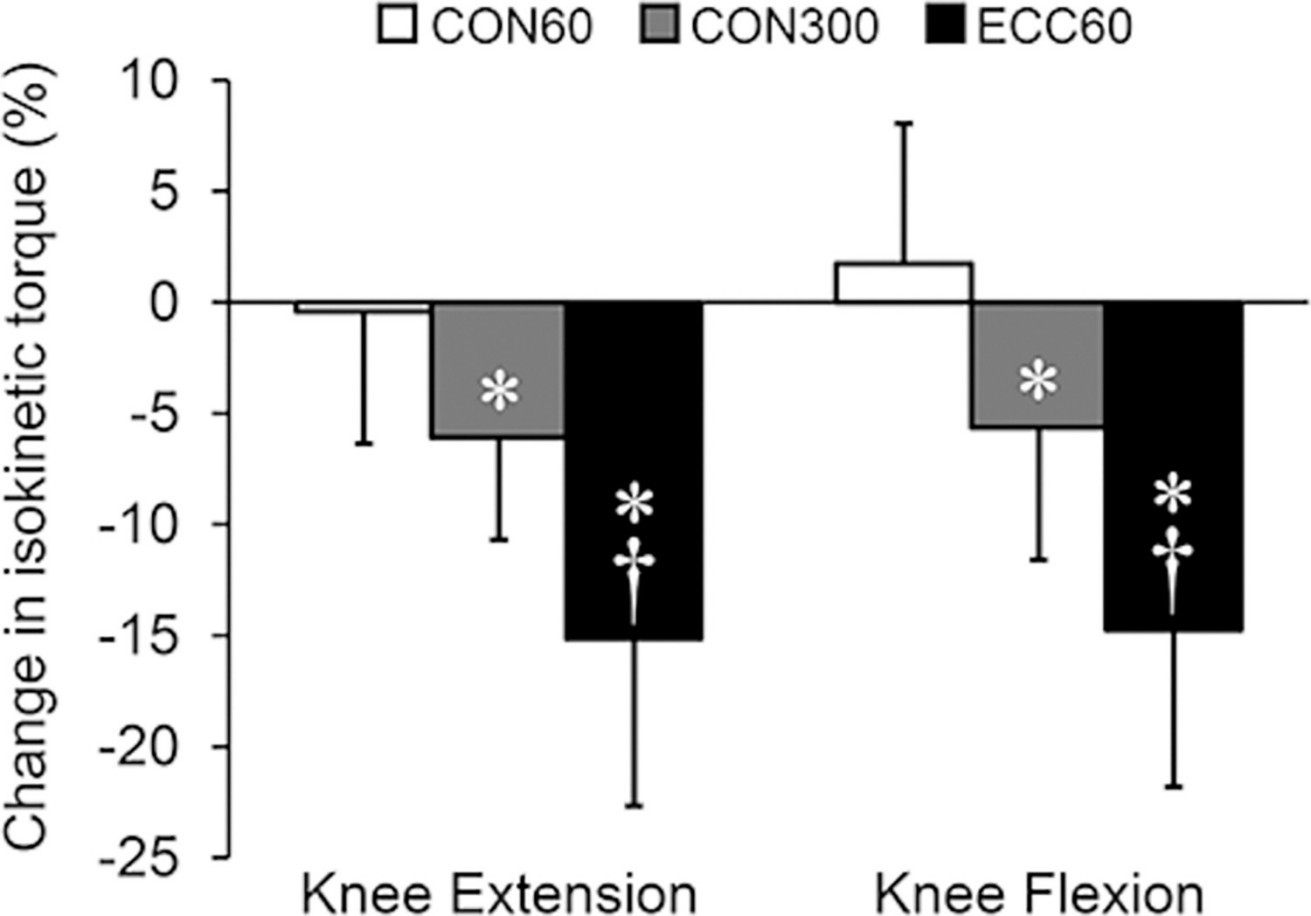
Percent change in isokinetic knee extension and flexion torque induced by two weeks of training cessation. CON60: concentric contraction at angular velocity of 60°/s, CON300: concentric contraction at angular velocity of 300°/s, ECC60: eccentric contraction at angular velocity of 60°/s, *Significantly different from CON60, †Significantly different from CON300.

Table 2 shows descriptive data on isokinetic knee extension and flexion torque of CON60, CON300, and ECC60 before and after the two weeks of training cessation. There were no significant differences in CON60 torque in either knee extension or flexion, whereas CON300 and ECC60 torques were significantly reduced in both knee extension and flexion. For the magnitude of changes in isokinetic torque (Fig 2), two-way ANOVA revealed a significant main effect of condition (CON60, CON300, ECC60) (P < 0.001, partial η^2^ = 0.706, observed power = 1.000), with no significant interaction of direction (extension, flexion) × condition (P = 0.618, partial η^2^ = 0.039, observed power = 0.121) or main effect of direction (P = 0.252, partial η^2^ = 0.108, observed power = 0.198). Post-hoc tests demonstrated significant differences with large effect sizes between CON60 (-0.7 ± 5.3%), CON300 (-5.9 ± 4.4%), and ECC60 (-15.0 ± 7.2%) (CON60 vs. CON300: P = 0.013, Cohen’s d = 1.068; CON300 vs. ECC60: P = 0.001, d = 1.524, ECC60 vs. CON60: P < 0.001, d = 2.265).

**Table 2:** Knee extension/flexion strength before and after two weeks of training cessation.

	Before	After	95% CI of difference		P value	Cohen’s d
			Lower	Upper		
Isokinetic knee extension torque (N⋅m)						
CON60	116.3 ± 15.9	115.4 ± 13.9	-5.2	3.3	0.650	0.061
CON300	72.4 ± 12.0	67.9 ± 11.6	-6.9	-2.0	**0.002**	0.382
ECC60	133.1 ± 33.2	112.9 ± 29.7	-26.9	-13.5	**< 0.001**	0.642
Isokinetic knee flexion torque (N⋅m)						
CON60	91.1 ± 20.1	92.1 ± 18.2	-2.6	4.6	0.550	0.053
CON300	56.1 ± 11.6	53.1 ± 12.3	-5.1	-1.0	**0.008**	0.251
ECC60	102.0 ± 22.0	87.0 ± 20.0	-20.1	-9.9	**< 0.001**	0.714
Nordic hamstring knee flexion torque (N⋅m)						
Left	108.8 ± 16.5	97.7 ± 12.3	-15.8	-6.3	**< 0.001**	0.763
Right	105.7 ± 13.0	97.1 ± 9.7	-14.4	-2.9	**0.007**	0.750
Dominant (Left/Right = 6/7)	106.4 ± 15.0	98.2 ± 11.5	-13.7	-4.3	**0.003**	0.614
Non-dominant	108.1 ± 14.9	94.6 ± 12.6	-17.5	-6.8	**< 0.001**	0.979

CI: confidence interval, CON60: concentric contraction at angular velocity of 60°/s, CON300: concentric contraction at angular velocity of 60°/s, ECC60: eccentric contraction at angular velocity of 60°/s

**Fig 3:**
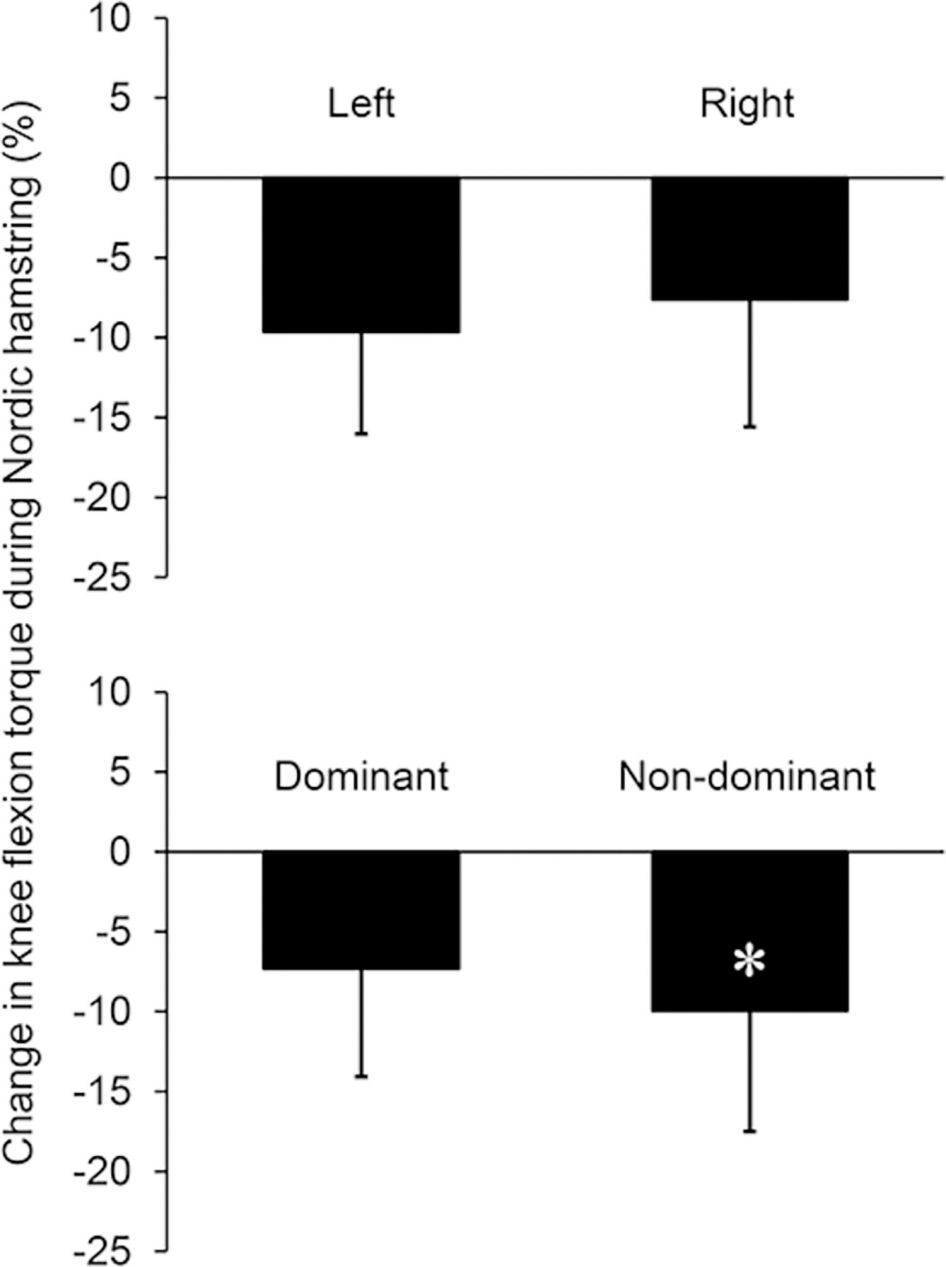
Percent change in Nordic hamstring knee flexion torque induced by two weeks of training cessation. *Significantly different from dominant leg.

**Fig 4:**
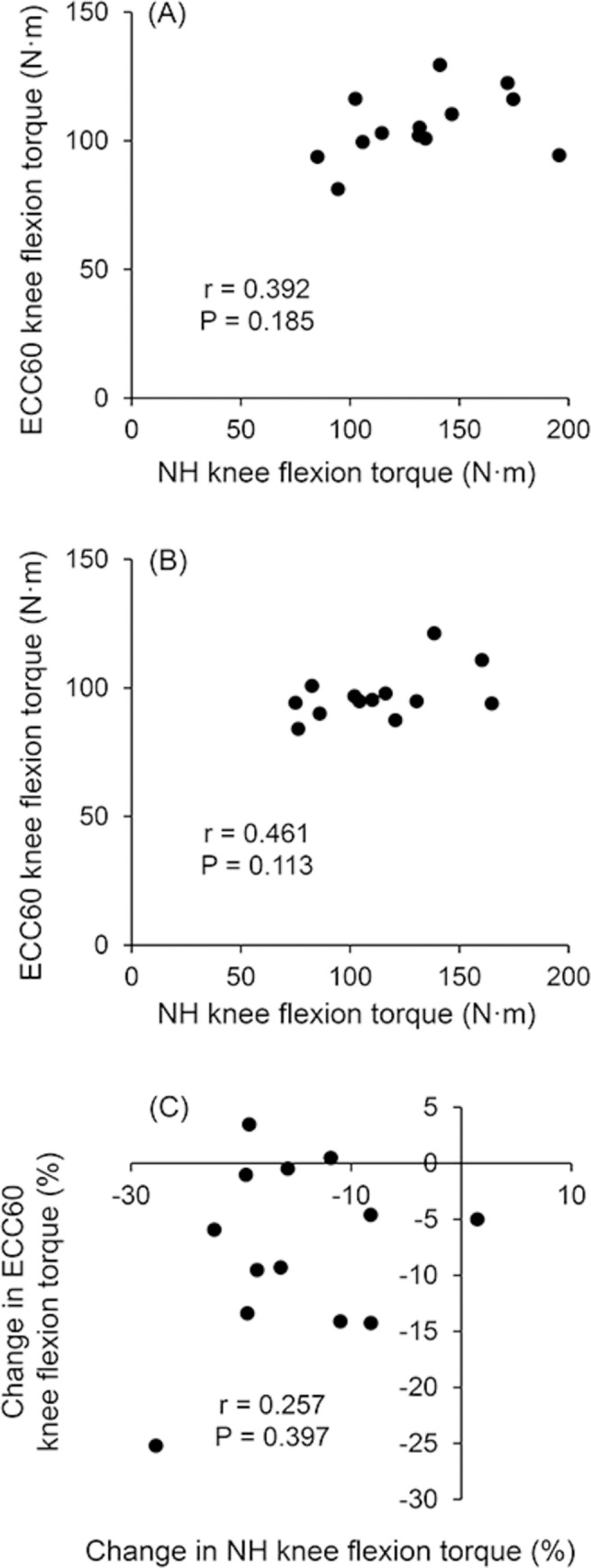
Relationship between isokinetic eccentric (ECC60) knee flexion torque and Nordic hamstring exercise (NHE) knee flexion torque of the right leg before the training cessation (A), between isokinetic eccentric knee flexion torque and NHE knee flexion torque of the right leg after the training cessation (B), and between the percent changes in isokinetic eccentric knee flexion torque and NHE knee flexion torque (C).

Knee flexion torques during the NHE before and after the two weeks of training cessation are shown in Table 2. Knee flexion torque was significantly reduced in both left and right legs and in both the dominant and non-dominant legs. The relative reduction of knee flexion torque during the NHE was significantly greater with a small effect size in the non-dominant than dominant legs (-9.9 ± 7.6% vs. -7.3 ± 6.8% for non-dominant and dominant legs, respectively, P = 0.039, d = 0.361), whereas there was no significant difference in the relative reduction between the left and right legs (P = 0.129) (Fig 3). For the knee flexion torque of the right leg, no significant correlations were found between ECC60 and NHE before (r = 0.392, P = 0.185) or after the training cessation (r = 0.461, P = 0.113), or between the relative reductions in ECC60 and NHE (r = 0.257, P = 0.397) (Fig 4).

## Discussion

The main findings of the present study were that 1) significant reductions in concentric strength after the two weeks of training cessation occurred only at high but not at low speeds; 2) the magnitude of reduction of muscle strength was more notable in slow eccentric contraction than in fast concentric contraction; 3) the effect of the two weeks of training cessation on muscle strength was comparable in knee extension and flexion in sprinters.

In the present study, slow (60°/s) isokinetic concentric torque in both knee extension and flexion remained unchanged, while isokinetic fast (300°/s) concentric and slow eccentric torque in both knee extension and flexion were reduced after the two weeks of training cessation. Although the segmental muscle mass of the left and non-dominant leg was significantly increased after the training cessation, the negligible differences and trivial effect sizes of < 0.2 [31] indicate the changes were virtually nil, which agrees with previous studies of strength-trained individuals [32]. Collectively, as proposed in a previous study [33], the training cessation-induced reductions in isokinetic fast concentric and slow eccentric torque might be attributed to neuromuscular activation.

Contrary to previous studies [7, 8], the present study showed that knee extension torque during fast concentric contractions was reduced after two weeks of training cessation. Knee extension torque during eccentric contractions was also reduced in the present study, which is in line with previous findings [7]. In order to attain “truly maximal” neuromuscular activation, much higher discharge rates of motor units are required during fast concentric contractions than during maximal voluntary isometric and slow concentric contractions: approximately 50 Hz for isometric or slow concentric contractions vs. 150–250 Hz or more for fast concentric contractions [34, 35]. It is unlikely, however, that untrained individuals who are less accustomed to performing fast concentric knee extension with maximal efforts are able to discharge motor units at such high rates [35]. Regarding neuromuscular activation during eccentric contractions, neuromuscular inhibition has been suggested to occur for untrained individuals, and indeed several studies have reported lower neuromuscular activation during eccentric contractions of knee extension than during isometric contractions, using electromyography or twitch interpolation techniques [9–11]. Although neuromuscular activation was not assessed in the present study, it is possible that in highly trained sprinters performing fast concentric and eccentric actions (including stretch-shortening cycle) of knee extension during sprinting, deficits in neuromuscular activation during fast concentric and eccentric knee extension are improved owing to long-term regular training. Taken together, we can conclude that neuromuscular activation during slow concentric contractions is high enough without training while sprinters are able to exhibit high neuromuscular activation during fast concentric and eccentric knee extension through prolonged training. Accordingly, knee extension torque during fast concentric and eccentric contractions is more likely to be affected by training cessation than that during slow concentric contractions in sprinters.

Fast concentric and eccentric isokinetic knee flexion torque was reduced after training cessation in the present study, whereas previous studies have reported that isokinetic knee flexion torque was not affected by short-term training cessation, regardless of concentric or eccentric contractions [6–8]. Similar to the aforementioned discussion on knee extension torque during fast concentric and eccentric contractions, the effect of short-term training cessation on isokinetic knee flexion torque may be dependent on the training background and specialization of the subjects: sprinters in the present study vs. powerlifters [7], soccer players, and endurance athletes in previous investigations. Although it is unclear how important knee flexion strength is for those athletes, the hamstring is crucial for superior sprint performance [13–15], resulting in reduced knee flexion torque after two weeks of training cessation in the present study. On the other hand, it is unclear why the reduction in isokinetic torque was greater in eccentric than fast concentric contractions in both knee extension and flexion. Future studies are warranted to explore the mechanism(s).

The present study observed that knee flexion torque during the NHE was reduced after two weeks of training cessation. The finding seems obvious, given the result that isokinetic eccentric knee flexion torque was reduced as mentioned above. However, we showed that knee flexion torque during the NHE was not correlated with isokinetic eccentric knee flexion torque at 60°/s measured by the isokinetic dynamometer in the pre-and post-tests, which is in line with previous studies [22]. Additionally, we found no significant correlation between the relative reductions induced by training cessation between the isokinetic eccentric knee flexion torque and knee flexion torque during the NHE. Such nonsignificant correlations could be due, at least in part, to the differences in knee joint angular velocity (60 deg/s for isokinetic vs. 180 deg/s for NHE), hip joint angle (flexed position for isokinetic vs. nearly neutral position for NHE), number of limbs used (unilateral for isokinetic vs. bilateral for NHE), the requirement of hip extension torque production (required only in NHE). Although the mechanisms remain to be further elucidated, the present findings suggest that eccentric knee flexion strength assessed by the isokinetic dynamometer test is highly likely to reflect different characteristics than that assessed by the NHE test. Thus, in future research, eccentric knee flexion strength and its training cessation-induced loss should be evaluated using both the isokinetic dynamometer and NHE tests.

The present findings have practical implications for preventing muscle function loss and re-training programs for improving impaired muscle function due to short-term training cessation. When athletes return to training or play after training cessation (e.g., because of injury, illness, post-season vacation, home confinement), they and their coaches may consider that their muscle condition is maintained based on one repetition-maximum or number of repetitions performed in high-load exercises. However, the present findings suggest that muscle condition assessed by slow concentric contractions can lead to missing significant reductions in fast concentric and slow eccentric muscle function caused by training cessation, increasing the risk of muscular injuries such as hamstring muscle strain. Especially, athletes, coaches, and athletic trainers should acknowledge that muscle function other than slow concentric muscle strength, especially eccentric muscle strength, can be dramatically reduced by training cessation as short as two weeks.

In addition to the lack of neuromuscular activation levels, as discussed above, one of the major limitations is the lack of a control group (i.e., subjects who did not experience the two weeks of training cessation). We recognize that with the current study design, it remains impossible to determine whether the changes are actually due to the two-week training cessation. For example, there is a possibility that the alignment of the rotation axis of the dynamometer with that of the knee joint varied between pre-and post-tests, which can result in the pre-to-post differences in muscle strength. However, this is unlikely because significant reductions after the two-week training cessation were observed for fast concentric and slow eccentric contractions but not for slow concentric contractions. Furthermore, the subjects were blinded to our hypotheses. Therefore, we believe that our interpretations and claims are credible. Additionally, while participants were instructed to maintain their normal daily activities without engaging in any physical training during the two-week period, their physical activities were not monitored during this time.

## Conclusions

Our results indicate that fast concentric and slow eccentric strength can be reduced in knee extension and flexion after training cessation as short as two weeks in sprinters. Notably, the reduction of eccentric muscle strength is notable. These findings suggest that athletes and their coaches should focus on regaining fast concentric and slow eccentric muscle strength after two weeks of training cessation due to off-season, COVID-related situations, injury, and illness.

## Supporting information

S1 Data set (XLSX)
